# Expectations in the field of the Internet and health: an analysis of claims about social networking sites in clinical literature

**DOI:** 10.1111/1467-9566.12203

**Published:** 2015-04-03

**Authors:** Nelya Koteyko, Daniel Hunt, Barrie Gunter

**Affiliations:** 1 School of Languages, Literature and Film, Queen Mary University of LondonUK; 2 Department of Media and Communication, University of LeicesterUK

**Keywords:** social media, e-health, metaphors, sociology of expectations

## Abstract

This article adopts a critical sociological perspective to examine the expectations surrounding the uses of social networking sites (SNSs) articulated in the domain of clinical literature. This emerging body of articles and commentaries responds to the recent significant growth in SNS use, and constitutes a venue in which the meanings of SNSs and their relation to health are negotiated. Our analysis indicates how clinical writing configures the role of SNSs in health care through a range of metaphorical constructions that frame SNSs as a tool, a conduit for information and a traversable space. The use of such metaphors serves not only to describe the new affordances offered by SNSs but also posits distinct lay and professional practices, while reviving a range of celebratory claims about the Internet and health critiqued in sociological literature. These metaphorical descriptions characterise SNS content as essentially controllable by autonomous users while reiterating existing arguments that e-health is both inherently empowering and risky. Our analysis calls for a close attention to these understandings of SNSs as they have the potential to shape future online initiatives, most notably by anticipating successful professional interventions while marginalising the factors that influence users’ online and offline practices and contexts.

## Introduction

Although the establishment of the World Wide Web dates back to the 1990s, the technology, its users and debates around them continue to change. As Wyatt (2004) points out: ‘There remains a great deal of interpretative flexibility regarding what it is, what problems it can solve, and what problems it may create’. A recent shift on this continually changing interpretative landscape is driven by the phenomenon of online networking, following the substantial growth in the use of social networking sites (SNSs) by individuals and organisations over the last 15 years (Madden and Zickuhr 2011). SNSs such as Facebook and Twitter are defined by boyd and Ellison (2007) as web-based services that allow users to create public profiles, pages and groups, articulate connections to other users, and ‘view and traverse their own and others’ lists of connections’. SNSs constitute one form of social media, a heterogeneous group of contemporary web applications that enable the creation, distribution and modification of user-generated content. The growth of social media thus represents an evolution from the original development of the web as a collection of static webpages with relatively limited opportunities for users to contribute content. Social media technologies, by contrast, facilitate the public interaction and collaboration of multiple users, with sites providing platforms through which this interaction is mediated. SNSs are among the most popular applications on the web and are represented by a diverse range of sites that vary in terms of their interfaces, memberships and how and with whom their users can communicate. This popularity is reflected in the increasing use of SNSs in the domain of health care (Hawn, 2009), where both individuals and organisations actively create SNS pages and groups to support different medical conditions (Farmer *et al*, 2009). With over one billion users (Facebook 2013), Facebook alone hosts 1068 pages established by US hospitals (Bennett 2011), while health-specific SNSs such as PatientsLikeMe allow users to establish profiles centred on longitudinal experiences of illness symptoms and treatments and connect with users in similar circumstances.

The features and affordances of SNSs have quickly become noticed in professional and policy circles. While some social scientists have recently begun to study the content and health-related practices on such sites (Lupton 2012), there is already a vast and growing body of clinical, psychological and information science literature discussing the existing and potential future applications of SNSs for health. In this article we aim to critically interrogate the understandings of SNSs and health that are articulated in the specific domain of clinical literature by drawing on concepts from science and technology studies (STS) and sociological research on e-health. The research investigates professional perspectives on SNSs for health by addressing the following question: how do these discussions construct and legitimise specific understandings of social networking technologies and health within this discursive domain? Specifically, our aim is to examine (i) models of user groups and their motivations and practices and (ii) the arguments and assumptions drawn upon to justify the use of SNSs in the healthcare context.

## The Internet and health

Sociological research on the phenomenon of e-health can be broadly divided into two stages. The first, coinciding with ‘the first age’ of the Internet (Haythornthwaite and Wellman 2002) focused on its novelty. These early studies tend to treat the Internet as separate from everyday life, leading to celebratory claims of its potential to challenge professional dominance and the biomedical orthodoxy (Henwood *et al*. 2003). Here, attention to structural constraints and embeddeness in corporate systems was sacrificed in favour of speculation about the contribution of the Internet to patients’ empowerment, where information and communication technology (ICT) itself was presumed to have a determinant impact upon society. In broader terms, this research echoed early theorisations of the information society where analytical priority was given to the allegedly inherent properties of ICTs. This set of views and principles, known as technological determinism (Webster 1995), represents the developments in ICT as an autonomous force that somehow transcends social constraints and interests, and in this way precludes considerations of human choice in our explanations of technology.

Some sociological research during the second stage strove to redress this imbalance by attending to structural inequalities and the institutionalised nature of medical information provision and sharing (Seale 2005). Following the ‘social shaping of technology’ approach (Grint and Woolgar 1997), which sees technological change as actively shaped by social, cultural and economic factors, the uses of ICT for health purposes have begun to be examined as part of wider socioeconomic conditions and institutional relationships and processes (Nettleton *et al*. 2005).

This article is part of a project that aims to contribute to this latter research by examining how the recent advent of social networking is interlinked and embedded in existing institutions and practices, and therefore is both constituted within and impacts upon social relations and cultural meanings (Sclove 1992). In particular, we draw on the notion of interpretative flexibility (Kline and Pinch 1999), which emphasises the dynamic and contingent process of articulating the meanings of technology. According to this constructivist position, new technologies invite different interpretations from different social groups, emphasising the fact that ‘the very core of technology, that which constitutes its working, is socially constructed’ (Bijker 1995).

## Data

The data were compiled through wide-ranging literature searches using the Science Direct, Scopus, Web of Knowledge, PubMed Central and UK National Institute for Health and Care Excellence Evidence Search databases. Searches were conducted during August 2013 using combinations of the following search terms: ‘social media’, ‘social network*’, ‘Facebook’, ‘health’ and ‘illness’.^1^ Searches were limited to texts published in English after 2005, a year that marked significant increases in registration to the popular MySpace and Facebook websites (boyd and Ellison 2007). The above searches generated 3190 hits that were screened to remove duplicates and establish relevance in terms of a focus on SNSs. Screening involved reading a publication's title, abstract and keywords and, where necessary, the main body to find whether it related to health and the uses of existing or bespoke web applications that fulfil boyd and Ellison's (2007) SNS criteria. Articles related to offline social networks, static health websites and discussion forums that did not meet boyd and Ellison's (2007) criteria were excluded at this stage. In order to focus specifically on texts by clinical professionals, articles were included if at least one of the named authors belonged to a healthcare organisation or worked in a healthcare-affiliated department of an educational institution. The resulting corpus consists of 80 articles, including primary research studies, systematic reviews and discussion papers as well as editorials, case studies and letters to clinical and professional journals.

## Methods and conceptual framework

A number of STS studies have explored how users consume, design, domesticate and resist technological development, as well as how users and uses are defined and configured (Weiner 2010) by different actors. These studies shed light on how the innovation process entails both ‘defining the identity of putative users, and setting constraints upon their likely future actions’ (Woolgar 1991). While Woolgar's work has illuminated how users are configured by designers, Oudshoorn (2012) has used the approach to study the co-construction of medical technologies as well as ICTs and their users. In this article, the focus on user configurations allows us to probe a range of claims made about SNSs as a relatively recent phenomenon in the e-health arena.

The framework of STS has developed an analytical arsenal for understanding the complex interactions between discourses of the future and the shaping of the present. Following Brown and Michael (2003) we examine the process of orchestration – that is, how different future scenarios are enabled or constrained, asking what kind of actions or possibilities are opened up or closed down by the key actors involved. Here, a close attention is paid to language use that can ‘mobilise attention, guide efforts and legitimate actions’ (Wilkie and Michael, 2009) by drawing on the analytical tools suggested by Dryzek (2005): the basic entities whose existence is recognised or constructed; assumptions about natural relationships between different entities; agents and their motives, and key rhetorical devices. This approach focuses both on the rhetorical composition of themes (Braun and Clarke 2006) and the underlying ideas and assumptions. Following the work by Wyatt (2004) and Segal (2009), we focus particularly on the metaphors used to describe SNSs in order to understand the perceptions and expectations of some of the actors involved in their shaping. Wyatt (2004), for example, critically examines transport and spatial metaphors that support references to the Internet as an ‘information superhighway’ and ‘cyberspace’. Such metaphors indicate the different ways in which the Internet and its services can be understood, with different figurative expressions foregrounding particular aspects of the topic (Koteyko 2014).

Crucially, the functions of such metaphors are not only explanatory but also rhetorical. As previous scholars have emphasised, metaphors have both cognitive and normative dimensions as they can ‘convey something about the future functions and technological configurations of the Internet, and they may also reveal the political assumptions and aspirations of those who deploy them’ (Wyatt, 2004). Metaphors are therefore often studied within the framework of the sociology of expectations (Coveney *et al*. 2009, Wallis and Nerlich 2005). In the case of emerging technologies such as SNSs, metaphors can shape future understandings of a platform and expectations of how it can be used.

## Analysis

The analysis was an iterative process as we re-read the articles and generated themes. Data coding was driven by our conceptual framework from STS and aimed to identify how SNS technology is configured in clinical research. To this end, coding focused on how SNSs are described, the individuals and groups presented as using (or potentially using) SNSs, and the reported functions of SNSs. The authors discussed their respective initial codes, which included particular forms of language (such as metaphor, comparisons and nominations) and emerging patterns in the data. This included examining, among other things, how SNS users were referred to (vulnerable users, information consumers, patients, audiences) as well as the practices attributed to them: seeking, producing, creating and controlling information as well as being overwhelmed by it or lost. Text extracts containing metaphors were systematically isolated and related expressions grouped together to enable the detailed study of expectations generated to steer social debate. To attest the metaphoricity of the identified tokens, two authors, well-acquainted with the metaphor-identification guidelines in cognitive linguistics, each read passages where the lexemes occurred to establish whether the use was metaphorical or literal and compared results. The interrater agreement was 97.3 per cent. Novel and conventional metaphors were both included, with domain incongruity as the major criterion for selection (Pragglejaz Group, 2007).

**Figure 1:**
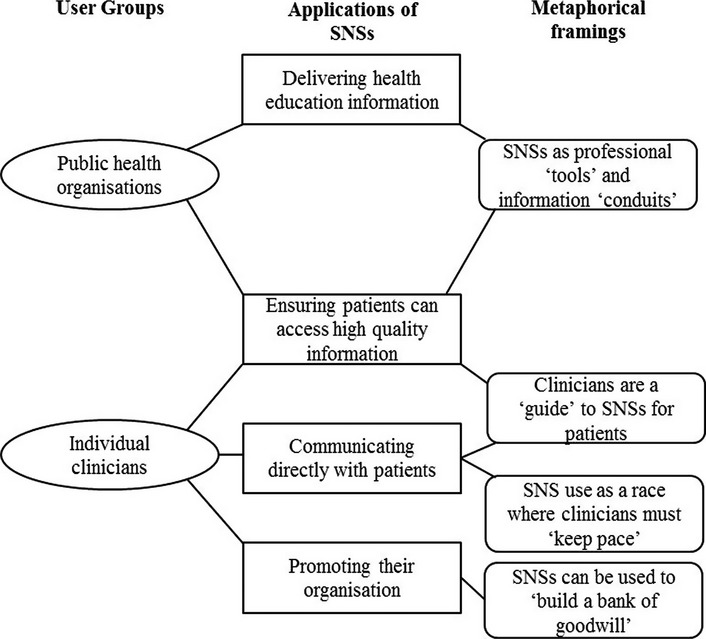
Applications and metaphorical framings of professional use of SNSs.

**Figure 2:**
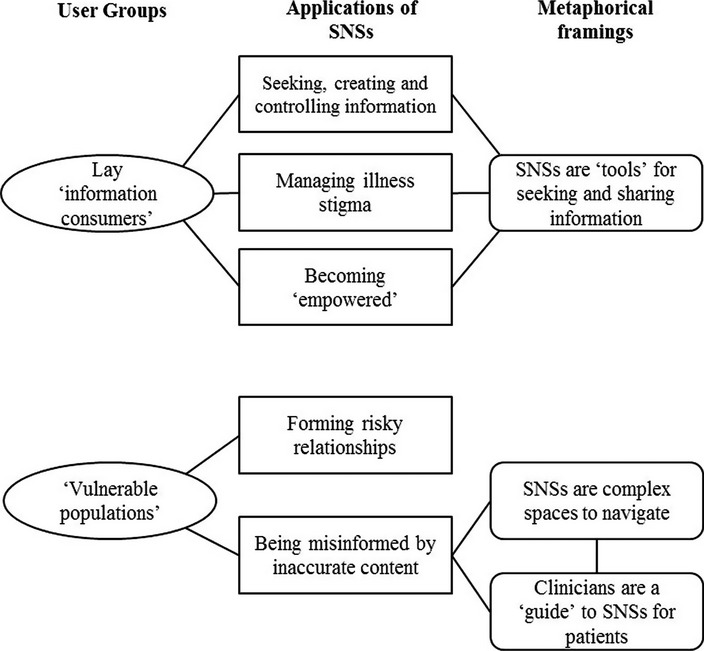
Applications and metaphorical framings of non-professional use of SNSs

The initial codes were organised into overarching themes (Braun and Clarke 2006) that identify consistent patterns and arguments which configure the role of SNS, groups of users, and their utilisation of SNSs for health. These themes are summarised in Figures 1 and 2. The following superordinate themes are discussed below: (i) SNSs as a means for public health organisations to deliver health promotion information to clinical and non-clinical populations; (ii) SNSs as a platform for individual doctors to communicate with their patients; (iii) SNSs as a means of connecting and ‘empowering’ non-professional users; (iv) SNSs as a venue for illegitimate information to be received and propagated.

### SNSs as a means for public health organisations to deliver health promotion information to clinical and non-clinical populations

Published articles that report medical studies involving a social networking intervention claim a role for SNSs in facilitating communication between researchers, health agencies, patients (McLaughlin *et al*. 2012) and the general public (Nguyen *et al*. 2013). A range of potential clinical uses is implied by claims that social media are sufficiently flexible and customisable to permit ‘widespread utility within the healthcare setting’ (Hamm *et al*. 2013). Central to this proposed utility is the communication brought about by social media technologies, making SNSs ‘a novel environment in which to deliver health promotion strategies’ (Gold *et al*. 2011: 1). Unfortunately, instead of harnessing the dialogical potential of SNSs, this communication is most commonly conceptualised via the conduit metaphor (Reddy, 1979),^2^ widely criticised in health communication research and public engagement literature for its unidirectionality (Condit *et al*. 2012, Koteyko, 2014, Wynne 1991). In this case, SNSs are conceptualised as a means to enable one-way provision of professionally authored health information to clinical and non-clinical populations:

Medical providers are now able to use SNH as a dissemination tool to ‘push information out’. (Sato and Costa-i-Font 2013)

This delivery of information is claimed to enable public health organisations to ‘inform, educate and empower people about health issues’ (Harris *et al*. 2013, Thackeray *et al*. 2012). In this way, social media are construed as having a potentially transformative effect on the lay population, fostering identities for members of the population as consumers who are willing and able to participate in contemporary models of patient-centred care and proactive self-management (Griffiths *et al*. 2012, Shaw and Johnson 2011). Here, the transformative utility of SNSs is underscored through the use of mechanical metaphors, most commonly conceptualising SNSs as a tool to provide health information and interventions to the public. The metaphor renders the use of SNSs by public health agencies and researchers as a physical process involving use of a medical tool and leveraging online content:

We must leverage the content, leverage the conversation, and leverage the good. (Timimi, 2012: 3)

Future research is needed to better understand how best to use social media as a tool for dissemination of health information to constituents and as a way to engage people living with and managing chronic disease. (Harris *et al*. 2013: 6)

[T]he image of social media as being the equivalent of a surgical scalpel – both are excellent tools but only if they are used appropriately and wisely! (Prasad, 2013)

The metaphor of social media as a surgical scalpel clearly situates SNSs alongside other routine clinical implements and legitimates their use for meeting professional goals. Also explicit in this claim is the notion of correct and incorrect professional uses of social media. As well as ensuring that patients have access to medically accurate information, Prasad (2013) notes the potential for breaches of patient confidentiality to occur through SNSs communication. In a similar vein, other authors refer to the provision of information via SNSs as a means to counterbalance harmful content in the form of private advertising and medically incorrect messages produced by patients (Freeman and Chapman 2008, Jones *et al*. 2013). Interventions into SNSs by researchers and public health organisations thus take the form of risk management of the online environment, both in terms of moderating the chances of lay SNSs users encountering medically unsupported content and minimising the chance of risky offline behaviour as a result of being misinformed about health issues.

### SNSs as a platform for individual doctors to communicate with their patients

Alongside research articles reporting SNSs interventions, articles that review the existing and expected use of SNSs by individual professionals configure individual healthcare providers as legitimate users of SNSs. In keeping with their use by health organisations above, SNS applications are identified as a means to communicate directly with patients. Such communication is envisioned as enabling a new, more personalised relationship between clinicians and their patients and encouraging patients to be more involved in their own care (Shaw and Johnson 2011). Here, SNSs represent a form of adjunct prescription from professionals who encourage their patients to use social media to learn more about their condition (Farmer *et al*. 2009).

The imperative for individual clinicians to use SNSs is encoded through the metaphor of clinical practice as a race in which healthcare providers must keep pace with the effects of social media (Antheunis *et al*. 2013), lest a gap open up between patients and providers (Eytan *et al*. 2011). Obligations on clinicians are also articulated more literally through an invocation of their professional responsibility to understand technology (Farmer *et al*. 2009) and claims that physicians must and need to adapt to the increasing use of SNSs by patients (Grover 2010, Timimi 2012). In parallel with the discussion of public health agencies, a central aspect of this putative responsibility is to manage the risks created by patients’ use of SNSs for their health. Hence Mousiolis *et al*. (2012) argue that doctors should guide patients towards particular health pages while Farmer *et al*. (2009) recommend pointing patients in the direction of SNSs.

A final SNSs activity suggested for this user group is promotion. This use is founded upon the generic description of SNSs as a platform that enables mass communication both with other healthcare professionals and with patient-consumer audiences. This allows SNSs to fulfil the role of a marketing resource with which clinicians can build a bank of goodwill via the networked dissemination of favourable content:

[E]ach impression [i.e. Tweet] equals a deposit to a bank of good will…. If the physician instead is identified as part of a Medical Group in their social media handle or profile, the deposit of good will goes to the organizational ‘account’ in addition to the individual ‘account’. (Eytan *et al*. 2011)

This framing of SNSs emphasises their potential for individuals to actively shape multimedia content online in order to create the desired public image for themselves and their practice. While not extensively discussed in the collated literature, the presupposition of an organisational marketing strategy imagines healthcare providers as competitors in a healthcare marketplace who promote themselves to potential stakeholders through SNSs. Aside from the health-promoting use of SNSs noted above, therefore, SNSs are constructed here as a vehicle for clinicians to meet professional objectives that are largely unrelated to the direct provision of healthcare. This imagined use of SNSs may well reflect the private US healthcare system in which these clinical authors operate. Nevertheless, the discourse of health consumerism underpinning this use is not limited to US contexts and is also evident in the discussion of non-professional users of SNSs for health.

### SNSs as a means of connecting and ‘empowering’ non-professional users

Numerous articles refer to individuals who are not healthcare professionals as users of SNSs for health. These non-professional users are identified both as members of clinical populations by studies sampling individuals with specific diagnoses (Greene *et al*. 2011, McDonald *et al*. 2013) and as members of a health-conscious public who seek out online information related to, among other things, nutrition, weight loss, sexual health and sun protection. This representation centres on the assumption of an active role for non-professional SNS users, which is encoded through the agency implicit in their framing as users, consumers, information consumers and health information seekers.

The distinction between traditional patients and contemporary health consumers is outlined by Lober and Flowers, who claim that ‘the main goal of being a patient is relief of illness, while the main goal of being a consumer is the efficient use of resources to meet personal goals’ (2011). In contrast to patients, therefore, users of SNSs for health are imagined as rational actors who utilise web platforms to fulfil an outcome-oriented strategy. This group of non-professional users is typically identified as seeking information and advice related to health conditions and behaviour, and the tool metaphor used in accounts of professionals’ use of SNSs also pervades descriptions of these lay users:

The Internet has been a tool for users and citizens to get more involved and empowered, and Web 2.0 tools take this to a new level. (Eysenbach, 2008)

Lefebvre and Bornkessel (2013) claim that consumers’ exchange of health information through SNSs is engendering a ‘new social health experience’ that contrasts with former individual experiences of health. The narrative here is one in which the uptake of social media signifies a radical transformation of established notions of patienthood, with users of online services now situated within connections to other users, family members, carers and healthcare professionals.

A similar argument is made by Lober and Flowers (2011), who see SNSs as enabling a significant departure from earlier e-health practices in which searches for information are replaced by engaging with a broad milieu of social actors. As they argue, the Internet changed ‘everything’ and ‘social media changes everything. Again’ (2011). Accounts of a newly social experience of health and illness brought about by SNSs stand in marked contrast to accounts from medical sociology, which emphasise that experiences of health have always been social in the sense of being understood in relation to prevailing social and cultural meanings (Crawford 1980). The purported social health experience may therefore arise less from the engagement of the lay population in novel practices; rather, it can be conditioned by the expanding and more visible networks articulated in the form of Facebook ‘friends’ and Twitter followers.

Clinical accounts attribute multiple benefits to non-professional users as a result of their active or passive involvement in SNSs for health. Chief amongst these is the notion of empowerment (Aujoulat *et al*. 2008). The process of empowerment and the precise role played in it by SNSs are seldom made explicit in the articles sampled, being instead attributed vaguely to opportunities for online learning and interactivity through SNSs (Korda and Itani 2013). More explicitly, Kamel Boulos and Wheeler state that SNSs’ increased opportunities for users to contribute online leads to the development of ‘collective intelligence’ and ‘reusable content’ (2007), while Merolli *et al*. claim that lay SNSs users’ support and sharing of information correlate with ‘empowerment outcomes’ (2013). Central to these claims is the principle that lay users are empowered by being active producers and curators of content on SNSs, which is, itself, said to lead to users’ increased involvement in healthcare decisions and the ability to fight negative assumptions about their condition (Gajaria *et al*. 2011).

The creation and presumed control of health discourses through social networking platforms provides another way in which this user group is configured as active and as distinct from those using websites with centrally authored content. That is, in addition to seeking information and establishing interpersonal connections, these users are presented as generating and sharing verbal, audio, visual and interactive health materials (Travers 2012) and are described as content creators and authors (Adams 2010, Gold *et al*. 2011). Echoing the tool metaphors above, such descriptions focus on the assumed technical mastery of SNSs by lay users – which, it is assumed, extends to users’ control over content:

Unlike mailed print media or electronic mail, social media lend themselves to a highly-customized user experience in which the user has control over the flow of information. (Travers 2012: 168)

In this way, SNSs consumers are configured as technology-enabled patients (Lober and Flowers, 2011), employing technical proficiency in the autonomous pursuit and control of empowering health information. At the same time, however, the proliferation of lay-authored health content on SNSs is also identified as problematic and as giving rise to an alternative narrative of lay SNS use, that of the vulnerable user.

### SNSs as a venue for illegitimate information to be received and propagated

Alongside the enabled consumer, a contrasting, disempowered lay user is clearly discernible in a significant proportion of current clinical SNSs literature and is framed by naming strategies such as patients, vulnerable populations and naive readers. Reflecting established professional concerns about patients’ consumption of web-based health information (Nettleton *et al*. 2005), the use of SNSs is presented as entailing several risks for this group. Most commonly, these risks centre on exposure to low-quality or unreliable information that leads to medically inaccurate beliefs and risky health behaviour (Vance *et al*. 2009). In addition to consuming misinformation, these lay users are said to divulge private information through SNSs, to be exposed to unregulated tobacco and pharmaceutical marketing (Liang and Mackey, 2011) and to fail to apply scientifically validated information to their own situations (Moorhead *et al*. 2013).

However, while the articles recurrently cite medically inaccurate information as a risk of using SNSs, responsibility for its consumption and production is often obscured. Hamm *et al*. (2013), for example, state that ‘the availability of misinformation is a risk’, rather than its production or uptake by patients. Similarly, Griffiths *et al*. (2012) attribute significant risks to SNSs and the ‘propagation of misinformation’:

[A] particular doctor or clinic could become the target of a wave of adulation or complaint or there might be a wave of people interpreting a pattern of bodily sensations as a sign of serious illness. Where these waves are relatively local rather than geographically dispersed, they have the potential to destabilise a local health care system.

The nominalised form of ‘propagation’ here obscures the active role played by SNS users in spreading content, meaning that agency is masked or attributed to the information itself. This contrasts with the active health consumers outlined in the previous section, while Griffiths *et al*.'s claim that social networking can lead to ‘herding’ presents lay users as passive and uncritical in their engagement with networking technologies.

Alternative accounts present lay users as more active but ultimately impaired in their SNS use. Jones *et al*. state that users seek out health information online but do so ‘despite being unable to verify the reliability of the information, its provenance, or underlying clinical evidence’ (2013). Similarly, Moorhead *et al*. (2013) argue that social media users are often unaware of risks and Weitzman *et al*. describe users of health-focused SNSs as ‘vulnerable populations who may poorly understand or discount privacy risks under conditions of countervailing need for information and support’ (2011). The emerging representation is of lay users who operate under either a knowledge deficit or contextual constraints that render them vulnerable to accepting harmful content and surrendering confidential information (Farmer *et al*. 2009).

D'Amato *et al*. (2012) postulate that adolescents are a particular subgroup of vulnerable SNS users. The authors argue that SNSs such as Facebook, rather than facilitating this group's exposure to medically unsound health information via SNSs, are in themselves a risk to adolescents as they replace real relationships and create opportunities for cyberostracism and resulting depression (2012). The role of SNSs in health is reconfigured here from a tool for gathering information that may be harmfully applied in users’ offline lives to a technology that affords new ways of establishing risky interpersonal relationships. Nevertheless, as with the accounts of lay users’ poor understanding of privacy and need for information, risks around SNSs are premised on the purportedly inherent vulnerability of this user group.

Vulnerable SNS users are positioned as partners for the individual clinicians, discussed above, who seek to guide their patients’ SNS behaviour. This relationship is underscored by a selection of complementary spatial metaphors that compel the intervention of medical professionals in SNSs. These metaphors construct social networks as complex traversable spaces and underlie descriptions of patients’ ‘aimless wandering’ (Kamel Boulos and Wheeler 2007), meeting a ‘barrage’ of online content (Travers 2012) and becoming ‘lost in the extra layers of information’ on SNSs (Adams 2010). These constructions provide the rhetorical ground for healthcare professionals to be positioned in the role of guiding their patients (Mousiolis *et al*. 2012) and helping ‘their patients’ to ‘find their way’ (Travers 2012).

The label ‘patients’ specifically identifies this user group as members of the clinical population to whom healthcare professionals can directly convey information during offline consultations. In contrast to the SNS consumer group, who purportedly act regardless of professional direction, the use of SNSs by vulnerable individuals is presented as an activity that must be professionally managed to prevent the patient coming to harm. The sampled articles therefore configure contrasting identities of non-professional users as both autonomous, discerning and technologically competent information consumers, and as patients rendered vulnerable by SNS use and needing guidance from professionals.

## Discussion

The analysis above has detailed the diverse imagined uses of SNSs for health by healthcare professionals, researchers and health organisations as well as by clinical and non-clinical members of the lay public. Descriptions of such uses and users contain numerous figurative expressions, most commonly tool and conduit metaphors. Thus, the use of SNSs by health professionals and organisations is framed as a skilled, controlled and largely one-way process in which professionals use SNSs to mediate changes to patients. In this process of information delivery, individual clinicians are constructed as guides who direct patients’ SNS use out of a sense of professional responsibility that encompasses patients’ health as well as their online privacy and SNS literacy. At the same time, non-professionals are portrayed as skilfully using SNS tools to access, create and control empowering health information, and also as consuming inaccurate content and engaging in risky communication.

The use of tool metaphors to frame social media may be specific to this domain of clinical articles about SNSs and their use to denote the skilful, successful manipulation of SNSs by healthcare professionals may well reflect the clinical backgrounds of the authors whose publications were included in our sample. Research published by, for example, psychologists or information scientists, may employ less positive framing devices to articulate clinicians’ use of SNSs. Indeed, representing healthcare as a process involving the manipulation of inert, mechanical patients by doctors using tools is an established metaphor in medical discourse (Hodgkin 1985). There are two problems inherent to the framing of SNSs as a tool, both stemming from the concomitant emphasis on the technology itself rather than the different contexts in which it is embedded. Accounts of SNSs as a powerful tool for healthcare are also challenged by recent review articles that cite the limited evidence for the effectiveness of health interventions involving social media (Chou *et al*. 2013, Hamm *et al*. 2013, Moorhead *et al*. 2013).

Firstly, the framing foregrounds the autonomy of individual users who are largely represented as being in control of the technology they use. This contrasts sharply with the corporate ownership of SNSs, that poses significant restrictions to users in terms of their access to and control of their accumulated data. For example, while Facebook users can view the data they create, the site does not guarantee that items posted will always be accessible. Users can currently download copies of their profiles and photos, but the company also reserves the right to use the personal data it collects. In this way, as McCown and Nelson (2009) put it, user activities are essentially trapped or ‘locked’ in the ‘walled garden’. Similarly, with few exceptions, the collated literature pays little attention to the corporate organisations operating in social media spaces that may monitor and exploit lay users’ contributions for commercial purposes and influence the content that users receive.

Secondly, the framing of healthcare as a process of mechanical manipulation also obscures the social and cultural contexts in which SNS users are embedded and the complex translation of SNS use into offline practices (Segal, 2009). This framing has been subject to longstanding critiques that argue that presenting the patient as a machine to be fixed by professional intervention backgrounds their identity and agency in the therapeutic process (Hodgkin 1985). For example, in reviewing the promotion of sexual health practices through SNSs, Gold *et al*. claim that future research should focus on ‘how to attribute success to the varying intervention components and website functionalities’ (2011). The emphasis is placed squarely on evaluating the technological affordances of SNSs for public health interventions rather than the offline behaviour of the target audience, their existing health and digital literacies and their situated use of SNSs. Similarly, although we identified several articles arguing that the use of professional health content on SNSs is dependent on the users’ offline contexts, the prevailing tendency is to privilege the delivery of information over the circumstances in which this information is interpreted and consumed. In addition to the tool metaphors, the unidirectionality of online healthcare interventions is further strengthened through the recurrent depiction of SNSs as a conduit for the provision and delivery of information to the public. Combined, these tool and conduit metaphors construe professional information as static and controllable, delivered and disseminated through SNSs channels.

In relation to their non-professional uses, SNSs are argued to take user involvement and empowerment ‘to a new level’ (Eysenbach, 2008: n.p.) by creating additional services with which health information can be accessed and reproduced. Closely linked to this representation is the frequent claim that SNSs can be used to engage social groups that are hard to reach through normal health communication media (Korda and Itani 2013). These include adolescents (Bull *et al*. 2012, Nguyen *et al*. 2013), individuals with mental health problems (Lehavot *et al*. 2012) and those from ethnic minorities (Shaw and Johnson 2011). Therefore, even while the typical non-professional user configured in clinical studies involving SNSs is young (Jelenchick *et al*. 2013, Moorhead *et al*. 2013), SNSs are claimed to be able to ‘compensate for peripherality’ by reaching the elderly, less well educated and physically disabled (Sato and Costa-i-Font 2013). As a result, SNSs are attributed a role in redressing health inequalities by allowing communication and information flow between health professionals and all areas of society.

This argument is made most explicitly by Laakso *et al.,* who claim that ‘[t]raditional barriers to accessing and implementing health information are largely alleviated through the unique capabilities offered by such social media platforms’ (2012). Demographic factors hindering healthcare are, they claim, largely dispelled through the social media, meaning that non-professionals using SNSs for health could potentially be any ethnicity, age or gender, or in any geographical location. This, in turn, supports an emphasis on information to be accessed and implemented by lay users as the salient component of SNSs. That is, since the lay user of SNSs is potentially *anyone*, priority is given to ensuring that information is accessible and reliable, with a concomitant de-emphasis of the complex personal contexts in which lay users may encounter and act upon health information found online (Segal 2009).

Offline contexts only partially come into picture in the peripheral discussions of non-users. Non-users of SNSs for health are nearly always identified as patients rather than professionals and are situated in relation to a range of offline characteristics. For example, Rozental *et al*. (2010) and Adams (2010) cite lower levels of education, income, digital literacy and broadband access as barriers to using SNSs for health reasons. In addition, McLaughlin *et al*. (2012) report that individuals who perceive a high level of social support in their offline social networks are less likely to seek peer support online. Here the non-user is embedded in an offline social milieu that militates against their desire for using SNSs for health, either by meeting their needs for support or their desire to avoid constructing a patient identity in their online interactions.

## Conclusions

Borup *et al*. (2006) assert that our present day understandings and expectations can manufacture the future just as well as they are recognised to be able to construct the past. In this article, we examined how the present and the future of web-based innovations are actively created through claims over potential applications in the domain of e-health. The analysis has enabled us to reveal the assumptions, visions, fears and closures nestled in particular understandings of the Internet and online social networking in the clinical literature, to trace them to a specific socio-historical context and social actors, and in this way to open them up to critical scrutiny (Wyatt 2004). As the audience of clinical journals includes other researchers, educators and policymakers, the representations of SNSs articulated in this domain have the potential to impact upon subsequent policy and interventions in this area.

Overall, the new affordances of SNSs have engendered claims that reiterate the empowerment discourse of early e-health research, in which the receipt of online content is said to stimulate users’ increased motivation and capacity for action in offline contexts as well as greater parity in clinical encounters. However, although today's multimodal, interactive and networked media spaces are indeed populated by active and creative users, the promissory rhetoric of consumer empowerment that has resurfaced in claims about SNSs backgrounds the fact that these users are still socially constrained. That is, assumptions that lay users are empowered by their consumption of medical information overlook the fact that users may seek alternative models of healing, may seek alternative models of healing and may lack the material and social resources to act upon information received online, and that their claims of lay expertise may be undermined by medical professionals (Henwood *et al*. 2003). These factors mean that the relationship between information and patient empowerment is by no means direct (Segal 2009) and is challenged by a culture of medical paternalism that is evident, not least, in the description of lay SNSs users as potentially vulnerable.

The uses of tool and conduit metaphors, together with representations of clinicians as guides, undermine claims that celebrate SNSs’ potential to empower lay users, engender many forms of knowledge and challenge medical expertise. When used in relation to professionals using SNS, these tool and conduit metaphors construe lay users as passive and suggest a one-way model of professional-patient communication. This may reflect the articles’ clinical authors privileging the professional's role in health communication in this context,^3^ while the health risks of SNSs are attributed solely to use by non-professionals. Indeed, just as SNSs become a new locus for discourses of patient empowerment in the clinical literature, constructions of vulnerable SNS users reiterate the medical profession's longstanding concerns over the proliferation of ‘low quality’ information online. Such reinvigorated ‘vulnerable patients’ rhetoric can fuel anxieties and potentially expand the remit of professional jurisdiction to encompass patients’ online behaviour (for example, Egan and Moreno 2011 already suggest the monitoring of university students’ SNS profiles for signs of their mental health problems). In configuring SNS for health, visions of its adroit professional use may well increase expectations of successful clinical interventions online, and mandate professional involvement as a means to safeguard ‘vulnerable’ patients. In line with the critique of early e-health discourses, sociologists need to pay attention to the role such claims can play in reinforcing the authority of biomedicine (Seale 2005) in a context where lay users may wish to pursue alternative therapeutics (Broom and Tovey 2008). More generally, the repetition of empowerment and risk discourses around SNSs signifies a lack of progression from early deterministic claims about the web, touch-screen kiosk and digital interactive television e-health platforms, which envisioned Internet technologies as a vehicle for delivering wider trends towards patients’ consumerism and self-management (Gunter 2005).

Aside from the binary rhetoric of hope and fear, visions of SNSs based on tool metaphors separate the technology from its users as well as from the contexts of its use. This representation of SNSs in isolation, as a tool adding certain features to online health-related activities (or impacting on them) is reductive, implying that the concepts of online participation, health and illness management and social media are both already known and unchanging. Taking into account the diverse and multiple factors that shape health-related behaviour we should instead be focusing on why, when and how these new technologies contribute to the everyday management of illness. Here the focus shifts from media to *mediation* (Livingstone 2008); the mediation of participation, identity and biomedical knowledge.

In line with existing analyses of SNSs in domains such as education, the study of how health-related phenomena are mediated is likely to uncover evolutionary rather than revolutionary change (Livingstone 2008). By considering different and multiple factors impacting on the use of SNSs for health-related activities, we will be able to examine how health and illness identities are reconfigured rather than completely transformed, with SNSs playing a role in mediating familiar activities rather than engendering brand new types of self-management behaviour and relationships. Examining how such processes work will help us understand SNSs and illness management in situ and inform education and support initiatives that respond to users’ online and offline practices and contexts.

## Notes

1 The asterisk functions as a wildcard filled by one or more characters. Searches for ‘social network*’ therefore retrieved results including ‘social network’, ‘social networks’ and ‘social networking’.

2 Only a few articles describe SNSs as fostering two-way communication between health agencies, researchers and non-professional SNSs users (Gold *et al*. 2011, Sajadi and Goldman 2011, Travers 2012, Villagran 2011).

3 The focus on clinical literature may have prevented us from identifying research on social support authored by social scientists.
